# Dysregulation of human *NEFM* and *NEFH* mRNA stability by ALS-linked miRNAs

**DOI:** 10.1186/s13041-018-0386-3

**Published:** 2018-07-20

**Authors:** Danae Campos-Melo, Zachary C. E. Hawley, Michael J. Strong

**Affiliations:** 1 Molecular Medicine Group, Robarts Research Institute, Schulich School of Medicine and Dentistry, Western University, London, ON Canada; 2 Department of Pathology, Schulich School of Medicine and Dentistry, Western University, London, ON Canada; 3 Department of Clinical Neurological Sciences, Schulich School of Medicine and Dentistry, Western University, London, ON Canada; 4 University Hospital, LHSC, Rm C7-120, 339, Windermere Road, London, ON N6A 5A5 Canada

**Keywords:** ALS, miRNA, Motor neuron, Neurofilament, *NEFM*, *NEFH*

## Abstract

Neurofilaments (NFs) are the most abundant cytoskeletal component of vertebrate myelinated axons. NFs function by determining axonal caliber, promoting axonal growth and forming a 3-dimensional lattice that supports the organization of cytoplasmic organelles. The stoichiometry of NF protein subunits (NFL, NFM and NFH) has to be tightly controlled to avoid the formation of NF neuronal cytoplasmic inclusions (NCIs), axonal degeneration and neuronal death, all pathological hallmarks of amyotrophic lateral sclerosis (ALS). The post-transcriptional control of NF transcripts is critical for regulating normal levels of NF proteins. Previously, we showed that miRNAs that are dysregulated in ALS spinal cord regulate the levels of *NEFL* mRNA. In order to complete the understanding of altered NF expression in ALS, in this study we have investigated the regulation of *NEFM* and *NEFH* mRNA levels by miRNAs. We observed that a small group of ALS-linked miRNAs that are expressed in human spinal motor neurons directly regulate *NEFM* and *NEFH* transcript levels in a manner that is associated with an increase in NFM and NFH protein levels in ALS spinal cord homogenates. In concert with previous observations demonstrating the suppression of *NEFL* mRNA steady state levels in ALS, these observations provide support for the hypothesis that the dysregulation of miRNAs in spinal motor neurons in ALS fundamentally alters the stoichiometry of NF expression, leading to the formation of pathological NCIs.

## Introduction

Neurofilaments (NFs) are unique neuron-specific intermediate filaments in vertebrates. They are highly dynamic structures that determine axonal caliber, promote axonal growth and organize the cytoplasm to form a stable 3-dimensional lattice that supports the organization of organelles and cytoplasmic proteins [1, 2].

NF subunit proteins (low, medium and high molecular weight neurofilaments; NFL, NFM and NFH, respectively) form homo- and hetero-polymers following a specific stoichiometry and tight spatiotemporal regulation. Conserving NF stoichiometry by controlling the levels of expression of individual NF subunits is critical for the maintenance of healthy neurons. Alterations of NF mRNA steady stoichiometry and the associated formation of neuronal cytoplasmic inclusions (NCIs) composed of NF proteins are neuropathological markers of degenerating motor neurons in amyotrophic lateral sclerosis (ALS), a progressive neurodegenerative disease [2–4]. Although the exact mechanism by which NF NCIs exert toxicity is unknown, it has been suggested that they alter the internal structure of axons and disrupt axonal transport, in addition to impairing NMDA-mediated calcium influx, compromising the survival of neurons [4, 5].

Post-transcriptional control is crucial for preserving NF subunit expression in neuronal homeostasis and also during axonal outgrowth in development and regeneration [6–8]. MiRNAs are evolutionary conserved non-coding RNAs that control the expression of the majority of the mammalian transcriptome and have been increasingly linked to neurodegenerative disorders. We and others have described a profound dysregulation of miRNAs in spinal cord and motor cortex of ALS patients [9–12]. We previously demonstrated that a selective group of these miRNAs directly regulate *NEFL* mRNA stability [9], and postulated that this dysregulation of miRNA expression would contribute to the selective suppression of *NEFL* mRNA levels observed in ventral lateral spinal cord motor neurons in ALS [13, 14]. Proper control of the levels of the NF triplet is critical because the backbone of the NF is mainly formed by NFL [15] and the stoichiometry of NFL/NFM/NFH (4:2:1) has to be carefully maintained [16]. The miRNAs responsible for regulating human *NEFM* and *NEFH* mRNA stability are however unknown. In this study we observed that a limited number of ALS-linked miRNAs that are expressed in spinal motor neurons directly regulate *NEFM* and *NEFH* mRNA levels, in a way that might explain the increase in NFM and NFH protein levels that we observed in ALS spinal cords and thus contribute directly to the formation of NF NCIs.

## Methods

### Tissue

Spinal cord samples from sALS patients (median age of death, 60.6 +/- 3.5 yrs) and age-matched, neuropathologically healthy control individuals (median age of death, 67.2 +/- 3.5 yrs) were used. All ALS cases were both clinically and neuropathologically confirmed using the El Escorial Criteria (World Federation of Neurology Research Group on Neuromuscular Disease, 1994). Written consent for autopsy was obtained from the next of kin at the time of death or from the patient antemortem in accordance with the London Health Sciences Centre consent for autopsy. ALS cases were genotyped and confirmed to have no known mutations in *SOD1, TARDBP, FUS* or expanded repeats in *C9orf72* (Table 1).

**Table 1:** Patient demographics

Cases	Gender	Age of symptom onset (years)	Symptom onset	Age of death (years)	Cause of Death
C1	F	–	–	62	Heart attack
C2	M	–	–	74	Stroke
C3	F	–	–	68	NA
C4	M	–	–	67	NA
C5	M	–	–	75	NA
C6	F	–	–	74	Leukemia
C7	M	–	–	68	Brain tumor
C8	F	–	–	53	Pneumonia
A1	F	58	NA	60	NA
A2	M	69	Upper/lower limbs	72	NA
A3	F	40	Bulbar	41	Systemic failure
A4	M	55	NA	61	Pneumonia
A5	M	64	Upper/lower limbs	67	Respiratory failure
A6	M	69	Respiratory symptoms	71	Respiratory failure
A7	F	63	NA	64	NA
A8	F	47	Bulbar	49	Respiratory failure

NA: Not available

### 3’RACE PCR, cloning and miRNA target prediction

*NEFM* and *NEFH* mRNA 3’UTRs were obtained using 3’RACE PCR. Briefly, TRIzol reagent (Thermo Fisher Scientific) was used for total RNA extraction from human spinal cord tissue. 3’RACE PCR was performed using SMARTer RACE 5′/3’ RACE Kit (Takara Bio. Inc., Clontech) and primers hNEFM_3RACE_F1D: 5’CACTTCACACGCCATAGTAAAGGAAGTCACC3′ and hNEFH_3RACE_F2: 5’GAGAAGGCCACAGAAGACAAGGCCGCCAAG3’ for *NEFM* and *NEFH* 3’UTRs, respectively. 3’UTR isoforms were cloned into pGEMT-Easy vector and sequenced. For luciferase assays, 3’UTRs were subcloned into pmirGLO vector between NheI and SalI sites and linked to the firefly luciferase coding region. Mutations in two nucleotides at the 3’end of each miRNA recognition element (MRE) within the *NEFM* and *NEFH* 3’UTRs were made using QuikChange Site-Directed Mutagenesis Kit II (Agilent) according to the manufacturer’s instructions. Mutations were carefully designed to ensure no changes were made in the secondary structures of the transcripts using the RNAFold WebServer (http://rna.tbi.univie.ac.at/cgi-bin/RNAWebSuite/RNAfold.cgi). Both TargetScan (http://www.targetscan.org/) and miRanda (http://www.microrna.org/microrna/getGeneForm.do) software programs were used to determine miRNAs with predicted MREs in either *NEFM* or *NEFH* 3’UTRs.

### miRNA extraction and real-time PCR

Total miRNA extraction using the mirVana miRNA isolation kit (Thermo Fisher Scientific) was performed from human ventral lumbar spinal cord using 5 controls and 8 ALS tissue samples according to the manufacturer’s instruction. Yield and purity of the miRNA solution was determined using spectrophotometry while RNA integrity was measured using a bioanalyzer instrument.

MiRNA extracts from the spinal cord of ALS patients or controls were reversed transcribed and then subjected to real-time PCR using the miRCURY LNA™ Universal RT microRNA PCR (Exiqon) and ExiLENT SYBR Green master mix (Exiqon), according to the manufacturer’s instructions. PCRs were performed using the 7900 HT real-time PCR system. Relative expression of miRNAs was normalized to miR-16-5p, a miRNA previously demonstrated to have the same expression in sALS and controls [9]. The analysis of the relative expression of candidate miRNAs between sALS and controls was done using the ΔΔCT method, where fold-change was calculated as 2^-ΔΔCT^. All experiments were run in triplicate and significance was determined using Student’s *t*-test.

### TaqMan real-time PCR

To examine the expression levels of *NEFM* and *NEFH* mRNA, total RNA extraction was performed on 6 ALS patient and 6 control lumbar spinal cord samples using TRIzol reagent (Ambion, Life Technologies). RNA samples were subjected to a cDNA synthesis reaction using the SuperScript IV VILO reverse transcriptase (Invitrogen, Thermo Fisher Scientific) in accordance to the manufactures instructions. Real-time PCR was done on the cDNA templates using the TaqMan Fast Advanced Master Mix and TaqMan Gene Expression Assays (Applied Biosystems, Thermo Fisher Scientific) targeting either *NEFM* or *NEFH*. Assays were performed in accordance to the manufactures instructions. TaqMan probes which either targeted *NEFM* or *NEFH* were designed with a FAM fluorophore. The expression of *NEFM* and *NEFH* were normalized to the expression of a reference gene (HPRT1), which was targeted by a TaqMan probe containing a VIC fluorophore. Changes in *NEFM* and *NEFH* mRNA expression between ALS patients and control subjects were determined using the ΔΔCT method, where fold-change was calculated as 2^-ΔΔCT^. Experiments were run in triplicate and determined to be significantly different using a Student’s *t*-test.

### Fluorescent in situ hybridization (FISH)

To ensure that the miRNAs of interest are expressed in human motor neurons, neuropathologically normal lumbar spinal cord from control subjects was examined for miRNA expression. Tissue sections were formalin-fixed, paraffin embedded (FFPE) and cut into 7 μm sections. Samples were UV treated overnight to reduce the lipofuscin-induced auto-fluorescent signal. FISH was performed as described previously (Planell-Sauger et al. 2010). LNA probes were designed with double DIG-labels that targeted the miRNA of interest (Exiqon). DIG-HRP secondary antibody, and Tyramide Signal Amplification (TSA) Systems tagged with a Cy3 fluorophore (PerkinElmer) were used to obtain a fluorescent signal of the miRNA target. Ventral horn of human lumbar spinal cord tissue was examined for positive staining within motor neurons using the Olympus FV1000 confocal microscope.

### Cell culture, luciferase assay and relative quantitative RT-PCR

HEK293T cells were maintained in Dulbecco’s Modified Eagle’s Medium (DMEM) containing 10% fetal bovine serum (FBS), at 37 °C with 5% CO_2_. HEK293T cells were plated on 96-well plates with a density of 10,000 cells/well 24 h prior to transfection. 100 nM of miRNA mimics (Thermo Fisher Scientific) and 3.47 fmol of pmirGLO containing *NEFM* or *NEFH* 3’UTR were co-transfected into the cells using Lipofectamine 2000 reagent (Thermo Fisher Scientific).

Luciferase assays and relative quantitative RT-PCR were performed 24 h post-transfection as was described previously [17]. Data show positive values as up-regulation and negative values as down-regulation. All experiments were run in triplicate, and significance was determined using a Student’s *t*-test or one-way ANOVA followed by Turkey’s post hoc test.

### Western blot

Total protein extraction from ventral lumbar spinal cord of 3 controls and 6 ALS patients was performed using NP40 lysis buffer containing proteinase inhibitors. Samples were sonicated, resuspended in loading buffer, denatured at 90 °C and run on an 8% SDS-gel. After transfer, the nitrocellulose membrane was probed with either mouse anti-NFM (1:1000; Boehringer Mannheim, 814–334), mouse anti-NFH (1:1000; Boehringer Mannheim, 814–342), or rabbit anti-GAPDH (1:5000; Abcam, ab9485) and later with HRP-secondary antibody (goat anti-mouse 1:3000, or goat anti-rabbit 1:5000; BioRad and Invitrogen, respectively). Relative protein expression of NFM and NFH were normalized to GAPDH expression levels. Student’s *t*-test was used to determine statistical differences in endogenous protein expression.

## Results

**Fig. 1:**
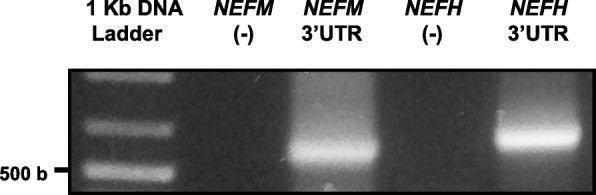
Single *NEFM* and *NEFH* mRNA 3’UTR variants are expressed in human spinal cord. 3’RACE-PCR was performed from spinal cord tissue samples of control individuals using specific primers to amplify *NEFM* and *NEFH* 3’UTRs. 3’UTRs were cloned and sequenced. One 3’UTR variant for each *NEFM* (486 nt) and *NEFH* (583 nt) transcript was observed in human spinal cord regardless of control or ALS origin of tissue. *NEFM* specific primer anneals to a region 40 nt upstream the stop codon

Considering that 3’UTR polymorphisms have been increasingly reported in the literature, we determined if 3’UTR variants of *NEFM* and *NEFH* mRNAs are expressed in human spinal cord. A single variant form of *NEFM* and *NEFH* 3’UTRs (486 and 583 nt, respectively) was detected in lumbar spinal cord control tissue (Fig. 1). Analysis of ALS patients showed no difference in the 3’UTR variants of *NEFM* and *NEFH* expressed in spinal cord compared to control samples (data not shown).

**Fig. 2:**
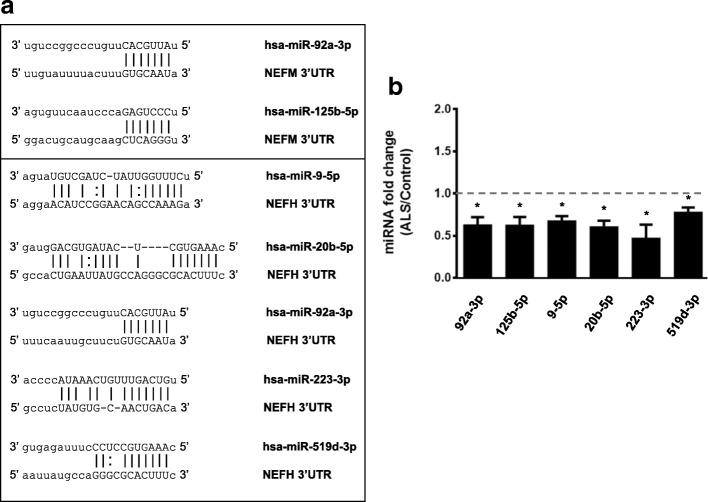
MiRNAs that have MREs in *NEFM* or *NEFH* 3’UTRs are down-regulated in the spinal cord of ALS patients. **a** MREs within *NEFM* and *NEFH* 3’UTRs of ALS-linked miRNAs. **b** Real-time PCR using SYBR green. Validation of differential expression in ALS versus control spinal cords of 6 miRNAs that have MREs in *NEFM* or *NEFH* mRNA 3’UTRs is shown. Experiments were performed in triplicate. Values below 1 indicate downregulation. Results are shown as mean ± SEM (Student *t*-test: **** *p* < 0.0001, *** *p* < 0.001, ** *p* < 0.01, * *p* < 0.05. MiR-9-5p, *p* = 0.0229; miR-20b-5p, *p* = 0.0230; miR-92a-3p, *p* = 0.0234; miR-125b-5p, *p* = 0.0412; miR-223-3p, *p* = 0.0215 and miR-519d-3p, *p* = 0.0232)

Prediction algorithms showed that *NEFM* and *NEFH* 3’UTRs have multiple MREs for different pools of miRNAs. However, for this study we only considered those miRNAs that we previously observed to be differentially expressed in ALS tissue versus controls using the TaqMan assay [9]. We performed real-time PCR using SYBR green of 40 miRNAs to validate differential expression of 6 miRNAs that have MREs in *NEFM* or *NEFH* 3’UTRs (Fig. 2a). Each miRNA, (miR-92a-3p, miR-125b-5p, miR-9-5p, miR-20b-5p and miR-223-3p and miR-519d-3p) showed significant down-regulation of expression in ALS spinal cord versus controls (Fig. 2b).

**Fig. 3:**
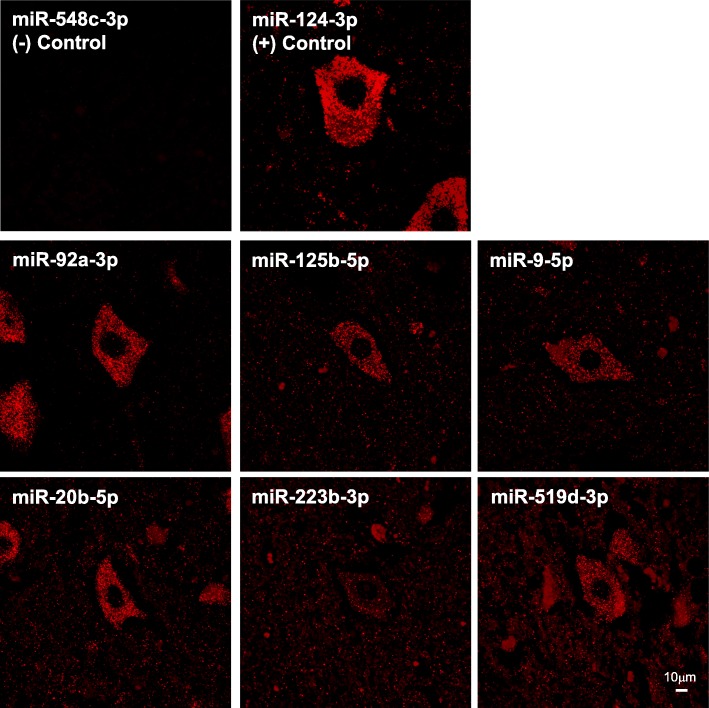
MiRNAs that have MREs within *NEFM* or *NEFH* 3’UTRs are expressed in motor neurons of human spinal cord control tissue. FISH was performed using FFPE control spinal cord tissue and LNA™-enhanced detection probes 5’-DIG and 3’-DIG labeled for miRNAs. Amplification was performed using anti-DIG-HRP and TSA Plus Cy3. MiR-548c-3p, which is not expressed in human spinal cord, was used as negative control. MiR-124-3p, which is known as highly expressed in neurons, was used as positive control

Next, we examined the neuronal expression of the group of miRNAs that potentially regulate *NEFM* and *NEFH* transcripts in human spinal cord motor neurons of control tissue through FISH. MiR-92a-3p is almost exclusively expressed in motor neurons of spinal cord. MiR-125b-5p, miR-9-5p, miR-20b-5p and miR-519d-3p showed higher expression in motor neurons than in other cell types within the spinal cord. MiR-223-3p showed similar expression in motor neurons and surrounding cells. MiR-548c-3p was used as a negative control and miR-124-3p, which is highly expressed in neurons, was used as positive control. In summary, we observed that the 6 ALS-linked miRNAs that are predicted to regulate *NEFM* and *NEFH* mRNAs are expressed in motor neurons of human spinal cord (Fig. 3).

**Fig. 4:**
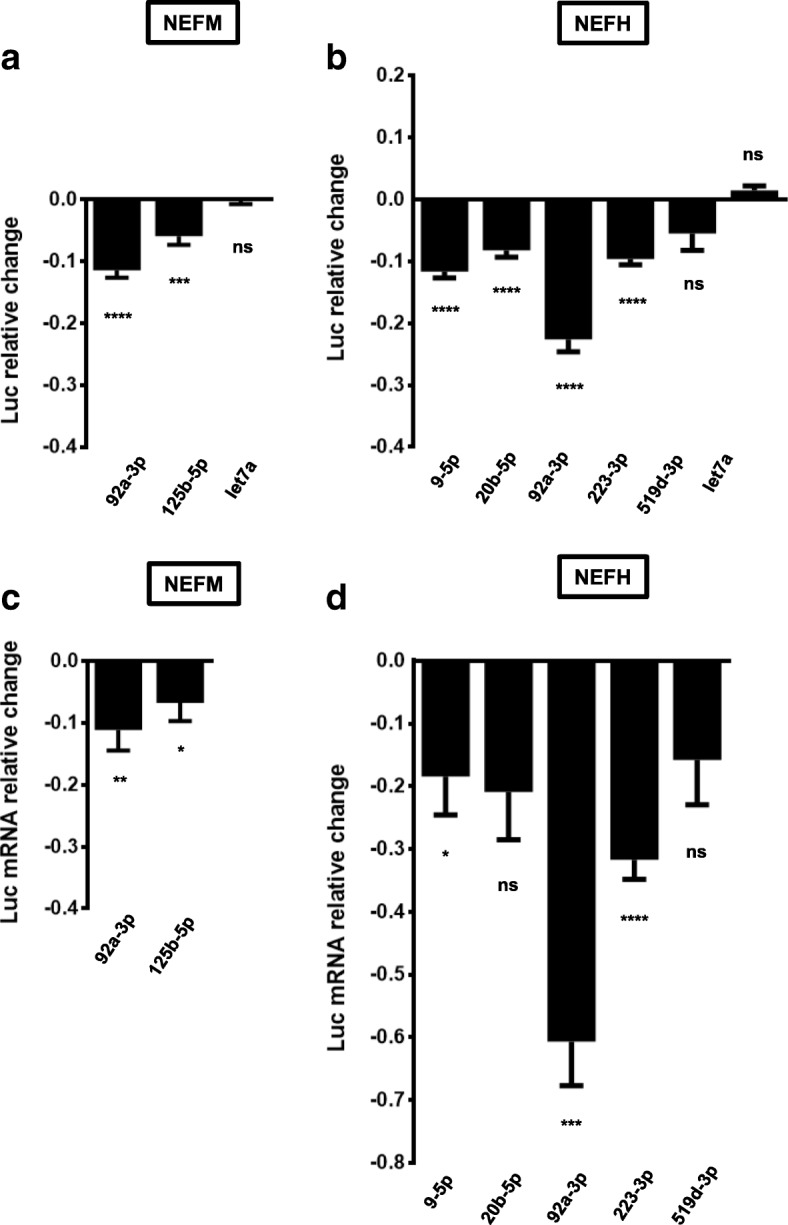
A group of ALS-linked miRNAs regulate a luciferase reporter linked to *NEFM* or *NEFH* 3’UTRs. HEK293T cells were co-transfected with a reporter plasmid containing *NEFM* or *NEFH* 3’UTRs and miRNA mimics. **a, b** Reporter gene assays were performed 24 h after transfection. Data are expressed as relative change and plotted in logarithmic scale. **c, d** Relative quantitative RT-PCRs were performed after RNA extraction, 24 h post-transfection. Data are expressed as relative mRNA level change and plotted in logarithmic scale. All experiments were performed in triplicate. Results are shown as mean ± SEM (Student *t*-test: **** *p* < 0.0001, *** *p* < 0.001, ** *p* < 0.01, * *p* < 0.05, relative to the pmirGLO vector control). Reporter gene assays: miR-92a-3p/NEFM, p < 0.0001; miR-125b-5p/NEFM, *p* = 0.0005; miR-let-7a/NEFM, *p* = 0.6015; miR-9-5p/NEFH, miR-20b-5p/NEFH, miR-92a-3p/NEFH and miR-223-3p/NEFH, p < 0.0001; miR-519d-3p/NEFH, *p* = 0.0723; miR-let-7a/NEFH, *p* = 0.0893. RT-PCRs: miR-92a-3p/NEFM, *p* = 0.0052; miR-125b-5p/NEFM, *p* = 0.0429; miR-9-5p/NEFH, *p* = 0.0431; miR-20b-5p/NEFH, *p* = 0.0518; miR-92a-3p/NEFH, *p* = 0.0003; miR-223-3p/NEFH, p < 0.001; miR-519d-3p/NEFH, *p* = 0.0903)

**Fig. 5:**
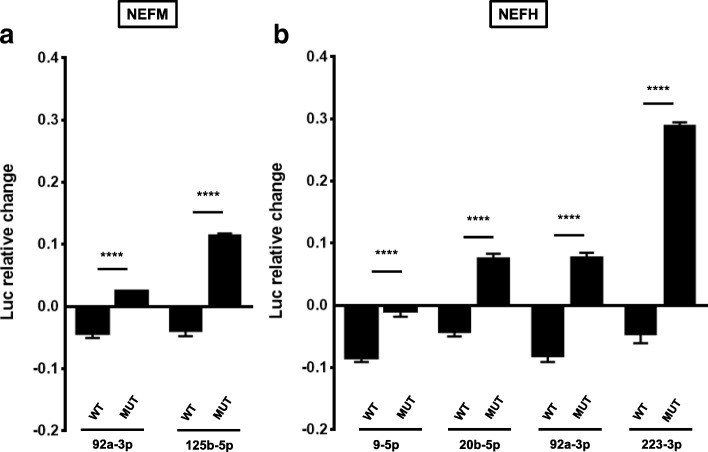
MiRNAs directly regulate luciferase transcripts linked to *NEFM* or *NEFH* 3’UTRs. HEK293T cells were co-transfected with a reporter plasmid containing mutant *NEFM* (**a**) or *NEFH* (**b**) 3’UTRs and miRNA mimics. Reporter gene assays were performed 24 h after transfection. Data are expressed as relative change and plotted in logarithmic scale. All experiments were performed in triplicate. Result are shown as mean ± SEM (One-way ANOVA followed by Tukey’s post hoc test: **** *p* < 0.0001, *** *p* < 0.001, ** *p* < 0.01, * *p* < 0.05, relative to the pmirGLO vector control). MiR-92a-3p/NEFM, miR-125b-5p/NEFM, miR-9-5p/NEFH, miR-20b-5p/NEFH, miR-92a-3p/NEFH and miR-223-3p/NEFH, *p* < 0.0001)

Functionality assays of these 6 miRNAs showed that miR-92a-3p and miR-125b-5p down-regulate the levels of a luciferase reporter linked to *NEFM* 3’UTR (Fig. 4a). MiR-9-5p, miR-20b-5p, miR-92a-3p and miR-223-3p down-regulate the levels of the luciferase reporter coupled to *NEFH* 3’UTR (Fig. 4b). We observed that most of these miRNAs also significantly down-regulate mRNA levels of the luciferase reporter bound to either *NEFM* or *NEFH* 3’UTR (Fig. 4c and d), which implies that miRNAs are dysregulating the stability of *NEFM* and *NEFH* transcripts. Reporter gene assay using *NEFM* or *NEFH* 3’UTR MRE mutants showed a decrease in the down-regulatory effect of each miRNA compared with the wild type, indicating that miR-9-5p, miR-20b-5p, miR-92a-3p, miR-125b-5p and miR-223-3p directly regulate *NEFM* or *NEFH* 3’UTRs stability (Fig. 5a and b).

**Fig. 6:**
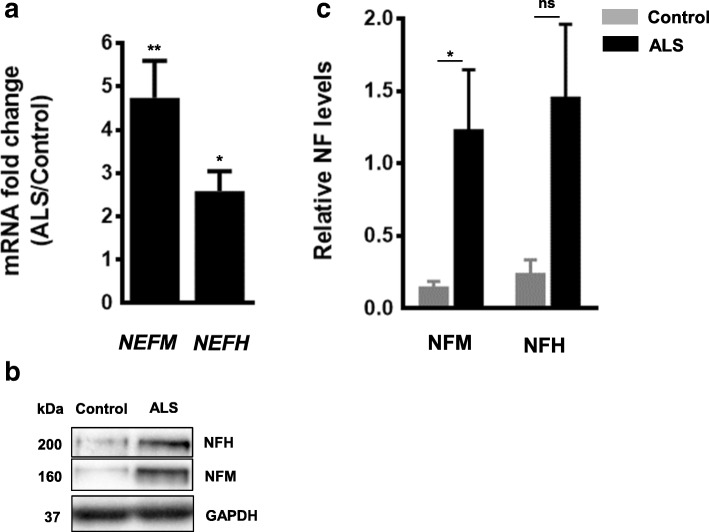
*NEFM* and *NEFH* transcript and protein levels are increased in spinal cord of ALS patients. (**a**) Real-time PCR using TaqMan and (**b**) Western blots were performed using ventral lumbar spinal cord samples of controls and ALS patients. (**c**) Quantification of Western blots in (**b**). Protein levels were normalized to GAPDH. Data was expressed as the mean ± SEM (Student *t*-test: **** *p* < 0.0001, *** *p* < 0.001, ** *p* < 0.01, * *p* < 0.05. Real-time PCR: *NEFM*, *p* = 0.0027: *NEFH*, *p* = 0.0239. Western blot: NFM, *p* = 0.0238; NFH, *p* = 0.4762)

Finally, considering the reduced expression of this group of 5 miRNAs in ALS spinal cords and the down-regulatory function they showed on *NEFM* and *NEFH*, we should expect an increase of *NEFM* and *NEFH* transcript and protein levels in ALS spinal cord tissue compared to controls. Consistent with this, we observed an increase in both *NEFM* and *NEFH* transcript and protein levels in ALS ventral lumbar spinal cords (Fig. 6a, b and c).

## Discussion

In this study we have shown that a small group of miRNAs that are dysregulated in the spinal cord of ALS patients directly regulate *NEFM* and *NEFH* mRNA stability and that this is associated with an increase in NFM and NFH protein levels in ALS spinal cord homogenates compared to neurological intact control spinal cord homogenates.

The post-transcriptional control of NF transcripts is critical for establishing, consolidating and maintaining normal levels of NF proteins. The stoichiometry of NF subunits has to be tightly controlled to promote axonal outgrowth, control axon caliber and avoid the formation of NF aggregates, axonal degeneration and neuronal death [4, 18]. The regulation of NF transcripts expression occurs at multiple levels. It has been reported that splicing of the last intron of *Xenopus NEFM* increases nucleocytoplasmic export of the transcript which allows for robust gene expression [19]. Another level of regulation is at the mRNA transport. One study observed that the mRNAs of each NF subunit are present and translated within intact and regenerating rat sciatic nerve, demonstrating that NF transcripts are transported through axons [20]. At the final stage of mRNA regulation, it has been shown that the RNA-binding protein HuB increases the translation of *NEFM* transcript [21].

mRNA stability is the regulatory process of NF transcripts most extensively studied in which multiple trans-acting factors participate. In mice, it has been shown that the RNA-binding protein p190RhoGEF stabilizes and that glycolytic isoenzymes aldolases A and C directly destabilizes *NEFL* mRNA [22, 23]. Our previous studies have shown that the stability of *NEFL* is regulated by ALS-associated RNA-binding proteins. Mutant copper/zinc superoxide dismutase (mtSOD1) and Rho Guanine Nucleotide Exchange Factor (RGNEF; the human homologue of p190RhoGEF) mediate the destabilization and TAR DNA binding protein 43 kDa (TDP-43) the stabilization of *NEFL* mRNA [24–26]. In addition, fused in sarcoma/translocated in liposarcoma (FUS/TLS), another ALS-associated protein, has been shown to bind to murine *NEFL*, *NEFM* and *NEFH* transcripts [27].

The most prominent mechanism of RNA mediated gene silencing involves the interaction of miRNAs with their target mRNAs in which most, but not all, interactions between the miRNA and MREs leads to a degradation of the mRNA. Previously, we and others have observed a massive down-regulation of miRNAs in ALS spinal cord [11, 12, 17]. We also showed that three miRNAs that are dysregulated in ALS, miR-146a*, miR-524-5p and miR-582-3p, regulate levels of *NEFL* mRNA [9]. In this paper we extended our study to miRNAs responsible for *NEFM* and *NEFH* post-transcriptional regulation. We created a list of miRNAs that are down-regulated in ALS spinal cord and that also possess MREs within *NEFM* and *NEFH* 3’UTRs. From the published literature, we observed that two miRNAs of this group (miR-9 and miR-125b-5p) were confirmed to be reduced in ALS spinal cord [11, 12]. We established that a small group of ALS-linked miRNAs (miR-9-5p, miR-20b-5p, miR-92a-3p, miR-125b-5p and miR-223-3p) directly down-regulate human *NEFM* and *NEFH* mRNA levels, an effect that is translated into a reduction of NFM and NFH protein levels within spinal cord homogenates. From this group of miRNAs that regulate *NEFM* and *NEFH* mRNA levels, only miR-9 has been reported to have a role in neuronal function. More specifically, by regulating several targets including *OC1, FoxP1, MAP1B,* and *MCPIP1,* miR-9 is critical for motor neuron development, function and survival [28].

As these group of miRNAs that regulate NFM and NFH are reduced in spinal cord of ALS tissue, we predicted that the net effect would be an increase of NFM and NFH protein levels in ALS-spinal cords. Several groups have shown that NFL, NFM and/or p-NFH levels are increased in biological fluids of ALS patients [29–33], but there are no reports of NFs protein levels in spinal cord tissue. In this study we showed that both NFM and NFH levels are increased in ventral lumbar spinal cord of ALS patients compared to controls. This observation is in agreement with the increased in *NEFM* and *NEFH* transcripts in ALS ventral lumbar spinal cord homogenates that we observed here using real-time PCR and reported previously using RNase protection assay [34].

A selective reduction of *NEFL* steady state mRNA levels in spinal motor neurons of ALS patients has been well documented [13, 14, 35], a finding that we have proposed is due to alterations in the expression of in *NEFL*-linked miRNAs [9]. In concert with the observations of this study, we hypothesize that in ALS spinal cords the sustained dysregulation in time of the expression of groups of miRNAs that control NF levels fundamentally alters the expression of all three *NF* transcripts in a manner that induces an alteration in the stoichiometry of the individual NF proteins, favoring the formation of pathological NCIs.

While this hypothesis supports the critical role of the alteration of miRNA expression in ALS, miRNAs alone are not the sole mediators of RNA stability. Indeed, understanding the fundamental relationship between alterations in RNA-binding proteins and how this interacts with alterations in miRNAs expression will be critical to understanding the process of perturbed RNA-mediated gene silencing which appears to lie at the core of a majority of ALS cases.

## Abbreviations

ALS: Amyotrophic lateral sclerosis
*C9orf72*: Gene encoding chromosome 9 open reading frame 72
FFPE: Formalin-fixed, paraffin embedded
FISH: Fluorescent in situ hybridization
FoxP1: Forkhead Box P1
FUS: Gene encoding RNA processing protein fused in sarcoma/translocated in liposarcoma
FUS/TLS: RNA processing protein fused in sarcoma/translocated in liposarcoma
GAPDH: Glyceraldehyde 3-phosphate dehydrogenase
*MAP1B*: Microtubule associated protein 1B
*MCPIP1*: Zinc finger CCCH-Type containing 12A
MRE: MiRNA recognition element
mtSOD1: Mutant SOD1
NCIs: Neuronal cytoplasmic inclusions
*NEFH*: High molecular weight neurofilament mRNA
*NEFL*: Low molecular weight neurofilament mRNA
*NEFM*: Medium molecular weight neurofilament mRNA
NF: Neurofilament
NFH: High molecular weight neurofilament
NFL: Low molecular weight neurofilament
NFM: Medium molecular weight neurofilament
NMDA: N-methyl-D-aspartate
*OC1*: Transcription factor onecut1
RACE: Rapid amplification of cDNA ends
RGNEF: Rho guanine nucleotide exchange factor
*SOD1*: Gene encoding Cu/Zn superoxide dismutase 1
*TARDBP*: Gene encoding TAR DNA binding protein 43 kDa
TDP-43: TAR DNA binding protein 43 kDa
TSA: Tyramide Signal Amplification
UTR: Untranslated region
